# Downregulation of NMI promotes tumor growth and predicts poor prognosis in human lung adenocarcinomas

**DOI:** 10.1186/s12943-017-0705-9

**Published:** 2017-10-12

**Authors:** Jingshu Wang, Kun Zou, Xu Feng, Miao Chen, Cong Li, Ranran Tang, Yang Xuan, Meihua Luo, Wangbing Chen, Huijuan Qiu, Ge Qin, Yixin Li, Changlin Zhang, Binyi Xiao, Lan Kang, Tiebang Kang, Wenlin Huang, Xinfa Yu, Xiaojun Wu, Wuguo Deng

**Affiliations:** 1 Sun Yat-sen University Cancer Center; State Key Laboratory of Oncology in South China; Collaborative Innovation Center of Cancer Medicine, Guangzhou, China; 2 Shunde Hospital, Southern Medical University, Foshan, China; 3 The First Affiliated Hospital of Dalian Medical University, Dalian, China; 4 Institute of Cancer Stem Cell, Dalian Medical University, Dalian, China; 5 Sun Yat-Sen Memorial Hospital of Sun Yat-Sen University, Guangzhou, China; 6 Cancer Center, Union Hospital, Tongji Medical College, Huazhong University of Science and Technology, Wuhan, China; 7 State Key Laboratory of Targeted Drug for Tumors of Guangdong Province, Guangzhou Double Bioproduct Inc., Guangzhou, China

**Keywords:** NMI, COX-2, NF-κB, p300, Lung cancer

## Abstract

**Background:**

N-myc (and STAT) interactor (NMI) plays vital roles in tumor growth, progression, and metastasis. In this study, we identified NMI as a potential tumor suppressor in lung cancer and explored its molecular mechanism involved in lung cancer progression.

**Methods:**

Human lung cancer cell lines and a mouse xenograft model was used to study the effect of NMI on tumor growth. The expression of NMI, COX-2 and relevant signaling proteins were examined by Western blot. Tissue microarray immunohistochemical analysis was performed to assess the correlation between NMI and COX-2 expression in lung cancer patients.

**Results:**

NMI was highly expressed in normal lung cells and tissues, but lowly expressed in lung cancer cells and tissues. Overexpression of NMI induced apoptosis, suppressed lung cancer cell growth and migration, which were mediated by up-regulation of the cleaved caspase-3/9 and down-regulation of phosphorylated PI3K/AKT, MMP2/MMP9, β-cadherin, and COX-2/PGE2. In contrast, knockdown of NMI promoted lung cancer cell colony formation and migration, which were correlated with the increased expression of phosphorylated PI3K/AKT, MMP2/MMP9, β-cadherin and COX-2/PGE2. Further study showed that NMI suppressed COX-2 expression through inhibition of the p50/p65 NF-κB acetylation mediated by p300. The xenograft lung cancer mouse models also confirmed the NMI-mediated suppression of tumor growth by inhibiting COX-2 signaling. Moreover, tissue microarray immunohistochemical analysis of lung adenocarcinomas also demonstrated a negative correlation between NMI and COX-2 expression. Kaplan-Meier analysis indicated that the patients with high level of NMI had a significantly better prognosis.

**Conclusions:**

Our study showed that NMI suppressed tumor growth by inhibiting PI3K/AKT, MMP2/MMP9, COX-2/PGE2 signaling pathways and p300-mediated NF-κB acetylation, and predicted a favorable prognosis in human lung adenocarcinomas, suggesting that NMI was a potential tumor suppressor in lung cancer.

## Background

Lung cancer is becoming the leading cause of cancer-related deaths worldwide [1, 2]. It is also the most common incident cancer and the leading cause of cancer death in China [3]. Non-small-cell lung cancer (NSCLC) accounts for more than 85% of lung cancer [4], while adenocarcinoma (AC) accounts for approximately 60% of all NSCLC and is the most frequently diagnosed subtype of NSCLC [5]. People with NSCLC can be treated with surgery, chemotherapy, radiation therapy, targeted therapy, or a combination of these. Although target therapy against epidermal growth factor receptor (EGFR) mutations and echinoderm microtubule-associated protein-like 4-anaplastic lymphoma kinase (EML4-ALK) rearrangements improved the prognosis in the last decade [6], mutations in EGFR are only present in 10–26% of NSCLC [7], and EML4-ALK rearrangements are only found in 4–5% of NSCLC [8]. Most patients are not associated with these mutations, and patients with advanced NSCLC are resistant to chemotherapy and radiotherapy. Therefore, improvements in lung cancer diagnostics and new treatments are urgently needed.

N-myc (and STAT) interactor (NMI) is a protein that interacts with NMYC and CMYC (members of the oncogene Myc family), and other transcription factors containing a Zip, HLH, or HLH-Zip motif [9]. The NMI protein interacts with all STATs except STAT2 and augments STAT-mediated transcription in response to cytokines IL2 and IFN-γ [9]. NMI is an IFN-γ inducible gene product that interacts with several key molecules in carcinogenesis such as SOX10 and TIP60 [10–14]. NMI may augment coactivator protein recruitment to some specific transcription factors, enhance the association of p300/CBP coactivator proteins with STAT1 and STAT5, and together with p300/CBP, augment IL2 and IFN- γ dependent transcription [9]. Previous studies demonstrated that NMI expression decreased in the progression of advanced invasive breast cancers [15–17], and loss of NMI expression promoted epithelial-mesenchymal-transition (EMT) [15]. It was also shown that restoring NMI expression inhibited tumorigenic and metastatic cell lines from anchorage independent and invasion related growth, and retarded tumor xenograft growth by inhibiting the Wnt/β-catenin signaling pathway and up-regulating Dkk1 [18]. In addition, NMI played a vital role in autophagy induction. Loss of NMI reduced the autophagy responsiveness and chemosensitivity of breast cancer cells [19]. Sun et al. identified NMI as an interactor of apoptin, a viral apoptosis inducing protein [20]. Nagel et al. discovered that the interaction between STAT5, NMI and N-myc repressed myocyte enhancing factor 2c and increased apoptosis in T cell acute lymphoblastic leukemia, suggesting that NMI might be involved in cancer cell specific apoptosis [21]. However, little is known about the function of NMI in lung cancer. In this study, we have found that NMI may promote apoptosis and inhibit cell growth and migration in lung cancer cells. Notably, we have shown that NMI regulates COX-2, an inducible enzyme that plays a vital role in carcinogenesis process.

COX-2 plays a key role in multiple pathophysiological processes including inflammation and carcinogenesis, as it influences apoptosis, angiogenesis, and invasion [22]. COX-2 is known to produce prostaglandin E2 (PGE2) that regulate tumor-associated angiogenesis, modulate the immune system, promote cell migration and invasion, and inhibit apoptosis, all of which promote cancer progression [23]. COX-2 is overexpressed in a wide range of human cancers, such as human cancers of colorectal [24, 25], breast [26], lung [27, 28], bladder [29–31], uterine cervix [32, 33], liver [34], pancreas [35, 36], prostate [37], skin [38], esophagus [39, 40] and stomach [41, 42]. COX-2 overexpression is positively related to poor survival [31, 43, 44], increased metastasis events, and invasive properties. In addition, COX-2 overexpression is associated with apoptosis resistance [45], and abnormal cell proliferation as a result of apoptotic cell death inhibition. In contrast, inhibition of COX-2 induces apoptosis signaling in various human cancers [46–48]. The COX-2 gene promoter contains an NF-κB response element as well as other cytokine-dependent (i.e., IL6) response elements [49]. COX-2 expression is transcriptionally controlled by the binding of activators such as NF-κB and coactivators such as p300 to the corresponding sites of its promoter [50–53]. However, it is unclear whether COX-2 expression is regulated by NMI in human lung cancer cells.

In this study, we investigated the role of NMI in the regulation of cell proliferation, apoptosis, and migration in human lung cancers, and also explored its underlying molecular mechanisms involved in PI3K/AKT, Erk/p38, MMP9/β-cadherin, NF-κB/COX-2/PGE2, and PARP/Bcl-2/Caspase3 signaling pathways. Tissue microarray and immunohistochemical analysis were also performed to assess the correlation between NMI and COX-2 expression. Moreover, we further identified the role of the transcriptional coactivator p300 in the NMI-mediated regulation of COX-2 expression and cell growth in lung cancer cells. Finally, we studied the clinical significance of the NMI/COX-2 signaling pathways in lung adenocarcinoma patients using Kaplan-Meier and multivariate survival analyses. The results from our study not only revealed the novel role of NMI in regulating lung cancer growth, but also provided a possibility for the development of NMI as a new potential tumor suppressor.

## Methods

### Cell lines and cell culture

H1299, HLF (HLF-a) and HBE cells were cultured in Dulbecco’s modified Eagle’s medium (DMEM) supplemented with 10% fetal bovine serum (FBS). A549 and H460 cells were cultured in RPMI1640 medium supplemented with 10% FBS. The cells were grown at 37 °C in an atmosphere of 5% CO2. All the cell lines were obtained from the American Type Culture Collection (ATCC, Manassas, VA).

### Plasmids

The NMI overexpression plasmid pEZ-Lv203-NMI (Catalog No.: EX-K2261-Lv203) and its control vector pEX-NEG-Lv203 (Catalog No.: EX-NEG-Lv203), NMI shRNA plasmid psi-LVRH1GP-NMI (Catalog No.: HSH022093-LVRH1GP) and its control vector psi-LVRH1GP (Catalog No.: CSHCTR001–1-LVRH1GP) were obtained from GeneCopoeia (Rockville, MD). The target sequence of NMI shRNA1 is 5′-gagtgcagtcatcacgttt-3′, and the target sequence of NMI shRNA2 is 5′-ctaggtcaacctcacatag-3′. A549 and H1299 cells were transfected with corresponding plasmids to overexpress or knock down NMI.

### Transfection

The cells (2 × 10^5^/ml) seeded in 6-well plates overnight were mixed gently 4 μg shRNA or plasmids and 10 μl of Lipofectamine 3000 (Invitrogen, Carlsbad, CA) in 250 μl opti-MEM (Gibco, Gaithersburg, MD) and incubated at 37 °C for 48 h.

### Lysate preparation from tumor tissues

Lung cancerous tumors and adjacent tissues were obtained from 20 lung adenocarcinoma patients who underwent surgery therapy at the First Affiliated Hospital of Dalian Medical University between 2014 and 2015. The surgery and the study had been approved by the medical ethics committee at the First Affiliated Hospital of Dalian Medical University, and the informed consents were obtained from patients in accordance with the Declaration of Helsinki and with institutional guidelines. The tissues (100 mg) were washed with PBS to remove blood, transferred to liquid nitrogen immediately and homogenized thoroughly with RIPA buffer with protease inhibitor. After incubation on ice for 30 min, these tissues were sonicated for 2 to 5 min at power of about 180 W. The lysates were centrifuged at 12,000 g for 20 min at 4 °C, and the supernatants were collected.

### Western blot

Protein lysates (40 μg) were separated by 4% to 12% SDS-PAGE, electrophoretically transferred to PVDF membranes, immunoblotted at 4 °C overnight with antibodies against NMI, p300 (Santa Cruz Biotechnology, Santa Cruz, CA), β-actin, COX-2, p-PI3K, PI3K p85 (Tyr458)/p55 (Tyr199), Akt, p-Akt (Ser473), p-PDK1, p-GSK3β, p110 β, P38, p-P38, ERK1/2, pPARP, pTyr202/Y204-ERK1/2, cleaved caspase-3, cleaved caspase-9, MMP2, MMP9, E-cadherin, B-cadherin (all purchased from Cell Signaling Technology, Beverly, MA), Bcl2 (Proteintech, Wuhan, China), followed by incubation with HRP-conjugated second antibody, and finally detected by enhanced chemiluminescence.

### PGE2 assay

Three days after transfection, cell culture media were collected. The amounts of PGE2 in the media were determined by Human Prostaglandin E2 (PGE2) ELISA Kit (Bluegene Biotech).

### Analysis of promoter activity

The promoter of human COX-2 gene (−891 to +9) was truncated to 6 different lengths. Each fragment was inserted into a luciferase reporter vector pGL3. Luciferase reporter assays were performed using the kit Dual-Luciferase® Reporter Assay System (Promega, Madison, WI).

### Cell viability assay

Cells were seeded into 96 well plates (4 × 10^3^ cells per well). Cell viability was measured by MTT assay 48 h after transfection.

### Apoptosis analysis

Cells were transfected with NMI overexpression or shRNA plasmid. After 48 h, cells were trypsinized, harvested, washed with PBS twice, and resuspended in Annexin V Binding Buffer at a concentration of 0.25–1.0 × 10^7^ cells/ml. 100 μl cell suspension was transferred to a 5 ml test tube, and 5 μl of APC Annexin V was added in, followed by the addition of 5 μl 7-AAD Viability Staining solution, per instructions of the Annexin V APC/7AAD staining kit (Keygen biotech, Jiangsu, China). Cells were pipetted up and down and incubated for 15 min at room temperature in the dark. Finally, 400 μl Annexin V Binding Buffer was added to each tube before the cells were analyze by flow cytometry (Beckman Coulter, Brea, CA). Apoptosis was measured in terms of the 7AAD-positive cells.

### Scratch assay

Cells were seeded in 6-well plates (4 × 10^5^ cells per well), and were grouped as (a) PBS control, (b) LacZ control, (c) NMI overexpression (d) control shRNA, (e) NMI shRNA1, (f) NMI shRNA2. 12 h after transfection, cells were scratched with sterile 200 μl tips, and photographed at 0 h, 48 h and 72 h.

### Confocal immunofluorescence

Cells were incubated on chamber slides in 6-well plates with or without NMI shRNA transfection. The samples were fixed with 4% polyoxymethylene for 30 min at room temperature, permeabilized with PBST (PBS with 0.2%Triton X-100), blocked with bovine serum albumin (BSA) for 30 min and incubated with NMI and p300 antibodies (1:200 dilution) overnight at 4 °C. After washed 3 times, cells were incubated with the fluorescein isothiocyanate and rhodamine conjugated secondary antibodies for 1 h. The nuclei were counterstained with 40,6-diamidino-2-phenylindole (DAPI). The images were taken by Olympus confocal laser scanning microscope.

### Immunoprecipitation (IP)

Nuclear protein (1 mg) was used for each IP. The proteins were pulled down with Protein A/G sepharose and then detected by Western blot analysis with specific antibodies.

### Animal study

Female nude mice (4 to 5 weeks old) were maintained in SPF laboratory Animal Central. All animals’ maintenance and procedures were carried in accordance with the National Institute of Health Guide for the Care and Use of Laboratory Animals, and passed through the training progress and approval by Animal Care and Ethics Committee of Sun Yat-sen University Cancer Center. Each nude mouse was injected with 2 × 10^5^ human A549 cells suspended in 100 μl PBS, subcutaneously near the axillary fossa. The mice were randomly divided into 5 groups (6 per group): (a) control plasmid (Ctrl); (b) NMI overexpression (NMI); (c) control plasmid + lipopolysaccharides (Ctrl + LPS); (d) NMI overexpression plasmid + LPS (NMI + LPS); (e) NMI shRNA + LPS (shRNA + LPS). As a transfection reagent, the complex of cholesterol-conjugated plasmid (10 μg) suspended in 100 μl saline were injected twice a week for 3 weeks. The dose of LPS for each mouse is 10 μg/kg, and LPS was injected twice a week for 2 weeks. Tumors volumes and body weights were measured every day. Tumor volumes were calculated as V = 1/2 (width^2^ × length). Mice were humanely sacrificed by euthanasia after treatment.

### Human tissue microarray

The human tissue micro arrays were purchased from Outdo Biotech Company (Shanghai, China). 75 cases from 75 lung cancer patients in total were arranged into two tissue array blocks.

### Human tissue immunohistochemistry

Slides were deparaffinized, rehydrated and then immersed in a target retrieval solution (pH 6) and boiled at medium baking temperature for three times with 10 min once in a microwave. After blocking the slides with 3% BSA, the sections were incubated with primary antibodies against NMI (Santa Cruz Tech., dilution 1:100) and COX-2 (Epitomics, dilution 1:100), and then with HRP-labeled anti-rabbit IgG secondary antibody. The specimens were counter stained with hematoxylin. A negative control was obtained by replacing the primary antibody with a regular rabbit IgG. The target-positive cells were counted in 3–4 different fields and photographed using an Olympus microscope, and the immunoreactions were evaluated independently by two pathologists blinded to the clinicopathologic information to ensure proper tissue morphology. Antibody staining intensity was categorized: no staining as 0, weak as 1, moderate as 2, and strong as 3. A five-scale system was used to categorize the percentage of cells strained: 0 (no positive cells), 1(<25% positive cells), 2 (25%–50% positive cells), 3 (50%–75% positive cells), and 4 (>75% positive cells). The score for each tissue was calculated by multiplying the intensity index with the percentage scale, and the range of this calculation was therefore 0–12. The median value of NMI scores was employed to determine the cutoff. Tumors with NMI scores lower or equal to the median were designated as “low expression”, whereas those with scores higher than the median were designated as “high expression”.

### TCGA Data analysis

The mRNA sequencing data of lung adenocarcinoma samples (*n* = 576) from The Cancer Genome Atlas (TCGA) were downloaded from UCSC XENA database, gene expression RNAseq (IlluminaHiseq pancan normalized) dataset. All samples were divided into three groups (i.e. high or H, red in heat map; medium or M, black in heat map; and low or L, green in heat map) based on raw gene expression values. NMI scores higher than 66.6% of the patients’ NMI scores were designated as “high expression”, between 66.6% and 33.3% of the patients’ were “medium expression”, and lower than 33.3% of the patients’ were “low expression”. All the samples were selected and used for analysis. The correlations of gene expression were accessed by Pearson correlation coefficient.

### Statistical analysis

Student’s t-tests were used to compare two independent groups of data. Chi-square tests were applied to analyze the correlation between NMI expression and clinicopathologic features of lung adenocarcinomas patients. Survival curves were constructed using the Kaplan-Meier method and were compared using the log rank test. Multivariate Cox proportional hazards analyses used “Enter” modeling to generate models predictive of outcome. Spearman correlation was used to explore the relationship between the abundance of NMI and COX-2. All calculations were performed with IBM SPSS 22 for Windows (SPSS, New York, NY, USA).

The results were presented as the mean ± SD of three independent tests. *P < 0.05, **P < 0.01, significant difference between the treatment and control groups.

## Results

### The expression of NMI was decreased in lung cancer cells and tumor tissues

**Fig. 1:**
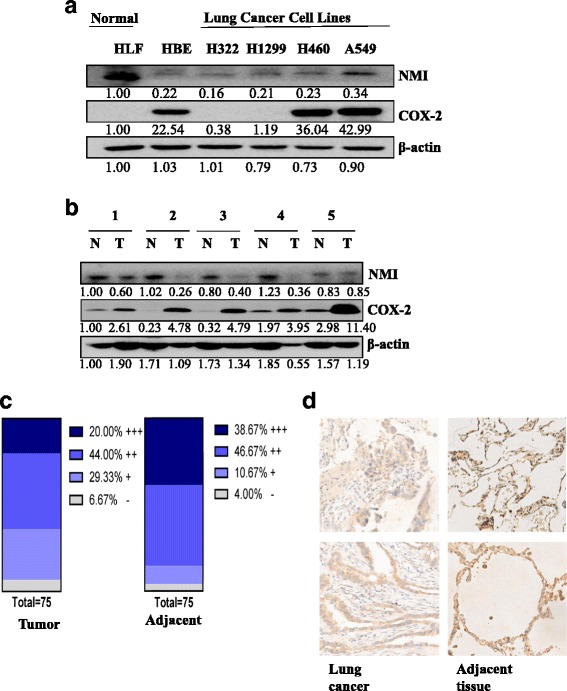
The expression of NMI was down-regulated in lung cancer cells and tumor tissues. **a** Western blot and quantitative analysis of NMI protein expression in human normal lung fibroblasts cells (HLF), immortalized human epidermal cells (HBE) and lung cancer cells (H322, H1299, H460 and A549). **b** Western blot and quantitative analysis of the expression of NMI protein in human lung cancer tissues (T) and adjacent normal lung tissues (N). **c** The distribution of lung adenocarcinoma patients with various levels of NMI expression in tumor tissues and adjacent normal lung tissues (*n* = 75). **d** Representative images of NMI immunohistochemical (IHC) staining in human lung adenocarcinoma tissues and adjacent normal lung tissues (10× magnification)

To analyze the expression of NMI in human lung cancer, we first detected the protein level of NMI in human lung fibroblasts cell line HLF, immortalized human epithelial HPV-16 E6/E7 transformed cell line HBE, and human lung cancer cell lines H322, H1299, H460 and A549 by Western blot and quantitative analysis. The result showed that NMI was expressed in all the tested cell lines, but lung cancer cells (H322, H1299, H460 and A549) had lower expression of NMI when compared with HLF cells (Fig. 1a). We next examined the expression of NMI and COX-2 in tumor tissues and their adjacent tissues from 20 lung adenocarcinoma patients using immunohistochemical and quantitative analysis. We found that 16 out of the 20 tumor samples (T) had lower expression of NMI and higher expression of COX-2 compared to their adjacent normal tissue samples (N). Five representative tumor (T)/normal (N) pairs were shown in Fig. 1b. This result was consistent with the observation in lung cancer cell lines H460 and A549 compared to normal cell line HLF (Fig. 1a). These data indicated that the expression of NMI may be negatively correlated with COX-2 levels in lung adenocarcinoma cases. We further investigated the expression of NMI in lung cancer tissues by immunohistochemistry assay. 75 tumor tissues and their adjacent normal tissues were tested, and NMI expression was observed in the nucleus and the cytoplasm of the cells in both tumor tissues and normal tissues. Among the 75 tumor tissues, 15 showed strong NMI expression, 33 moderate, 22 weak, and 5 negative. In contrast, among the 75 adjacent normal tissues, 29 showed strong expression of NMI protein, 35 moderate, 8 weak, and 3 negative (Fig. 1c). The representative images for NMI expression in lung cancer tissues and normal tissues were shown in Fig. 1d. These results suggested that NMI had lower expression in lung cancer cells and tissues.

### NMI inhibited proliferation and induced apoptosis of lung cancer cells

**Fig. 2:**
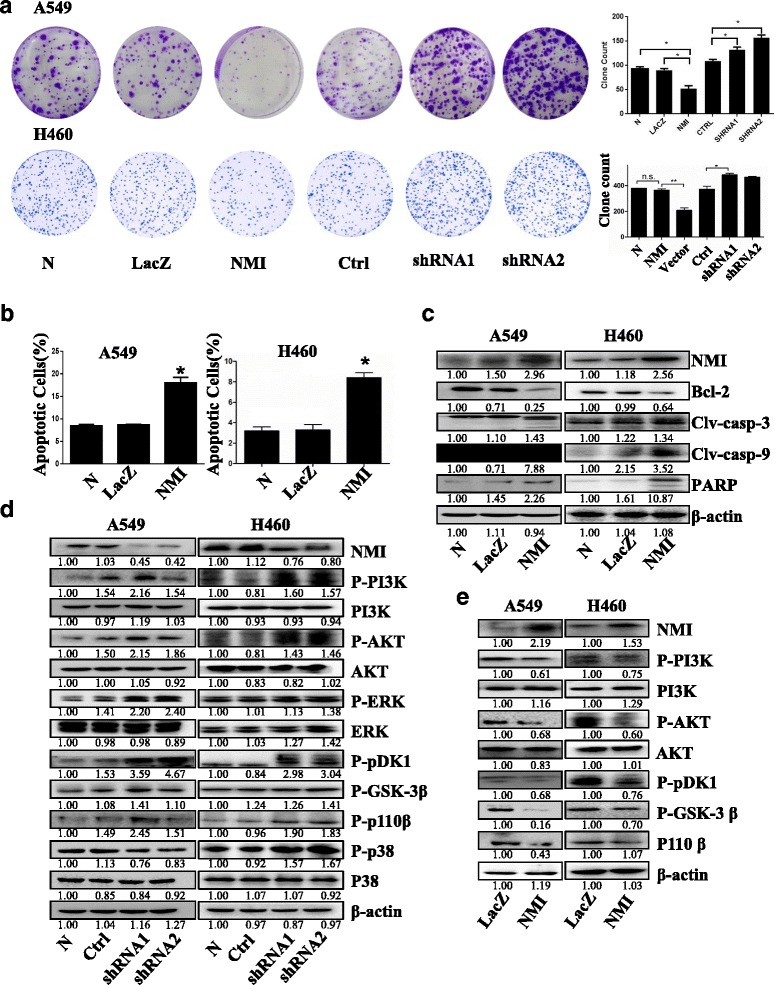
NMI inhibited clone formation and induced apoptosis in lung cancer cells. **a** Colony formation assay and quantitative analysis were performed with in A549 and H460 cells with NMI overexpression or knockdown. **b** FACS analysis was used to determine the relative percentage of apoptotic cells in A549 and H460 cells. **c** Western blot and quantitative analysis were used to detected the expression of Bcl-2, cleaved caspase-3/9 and phosphorylated PARP proteins in A549 and H460 cells at 48 h after transfection with NMI or control plasmids. **d** Western blot and quantitative analysis were used to detected the levels of phosphorylated PI3K, AKT, ERK, PKDK1, GSK-3β, p110β and p38 proteins in A549 and H460 cells at 48 h after transfection with NMI-shRNAs or control. **e** Western blot and quantitative analysis were used to detected the expression of the phosphorylated PI3K, AKT, pDK1, GSK-3β and p110β proteins in A549 and H460 cells at 48 h after transfection with NMI overexpression or control plasmids

The effect of NMI on lung adenocarcinoma cell proliferation was evaluated in lung cancer cell lines A549 and H460 by colony formation and quantitative analyses. NMI overexpression significantly inhibited the proliferation of both A549 and H460 cells. In contrast, knockdown of NMI remarkably promoted the proliferation and increased the clone volume of A549 and H460 cells (Fig. 2a). These results indicated that NMI played an important role in regulating lung adenocarcinoma cell proliferation. Next, the effect of NMI on apoptosis was investigated by Annexin V APC-7AAD staining-based FACS analysis. Overexpression of NMI significantly induced apoptosis in both A549 and H460 cells (Fig. 2b). We then examined the effect of NMI on the expression and activation of four key proteins in the apoptosis signaling pathway: Bcl-2, caspase-3, caspase-9, and PARP in A549 and H460 cells. Overexpression of NMI repressed the anti-apoptotic protein Bcl-2, and activated caspase-3, caspase-9 and PARP, which was indicated by marked increases in cleaved caspase-3, cleaved caspase-9 and phosphorylated PARP (Fig. 2c). Thus, these results indicated that NMI might control several aspects of apoptosis signaling. To further identify the potential molecular mechanisms by which NMI inhibited lung cancer cell growth, we analyzed the expression of a series of pro-survival proteins possibly affected by NMI by Western blot and quantitative analysis. The results showed that NMI knockdown in A549 and H460 cells dramatically enhanced the phosphorylation of PI3K, AKT, PDK1, GSK-3β and p110 β proteins, but did not change the expression of total PI3K, AKT proteins themselves (Fig. 2d). On the contrary, overexpression of NMI in A549 and H460 cells significantly inhibited the phosphorylation of PI3K, AKT, PDK1, GSK-3β, and p110 β proteins, without changing the levels of total PI3K and AKT (Fig. 2e). We also observed the up-regulation of the phosphorylation of ERK and p38 in the MAPK signaling pathway upon NMI silencing (Fig. 2d). These data suggested the possible involvement of PI3K/AKT/MAPK signaling pathways in NMI-mediated lung cancer cell growth inhibition.

### NMI inhibited lung cancer cell migration

**Fig. 3:**
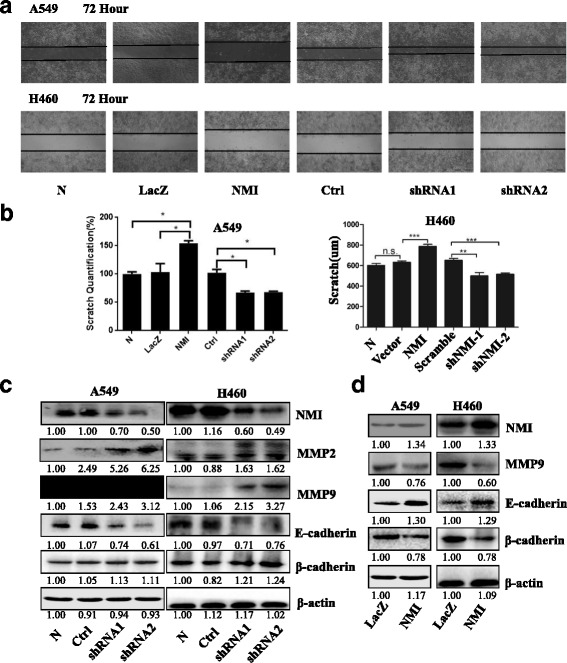
NMI inhibited lung cancer cell migration. **a** Scratch assay was used to detected the cell migration in A549 and H460 cells at 72 h after transfection with NMI overexpression/control plasmids or NMI shRNAs/control plasmids. **b** Quantification of scratch assay. **c**, **d** Western blot and quantitative analysis were used to detected the expression of MMP2, MMP9, E-cadherin and B-cadherin in A549 and H460 cells

We further investigated whether the NMI-mediated inhibition in lung cancer cells was associated with the impeded cell migration ability. We examined the effect of NMI overexpression or knockdown on the migration of A549 and H460 cells by scratch assay. The gap between cell layers made by a scratch was much wider in the NMI-overexpressed cells, but it was much narrower in the NMI-knockdown cells (Fig. 3a, b). Additionally, the expression of MMP2 and MMP9, two key molecules involved in cell migration, was significantly increased in NMI-knockdown cells. Meanwhile, the expression of E-cadherin, which plays a critical role in cell adhesion, was markedly down-regulated, whereas the expression of β-cadherin was unaffected (Fig. 3c). In contrast, in the NMI-overexpressed cells, MMP9 and β-cadherin was down-regulated, whereas E-cadherin was up-regulated (Fig. 3d). These results indicated that NMI played a vital role in the inhibition of cancer cell migration.

### NMI inhibited transcriptional activation of COX-2 and PGE2 production

**Fig. 4:**
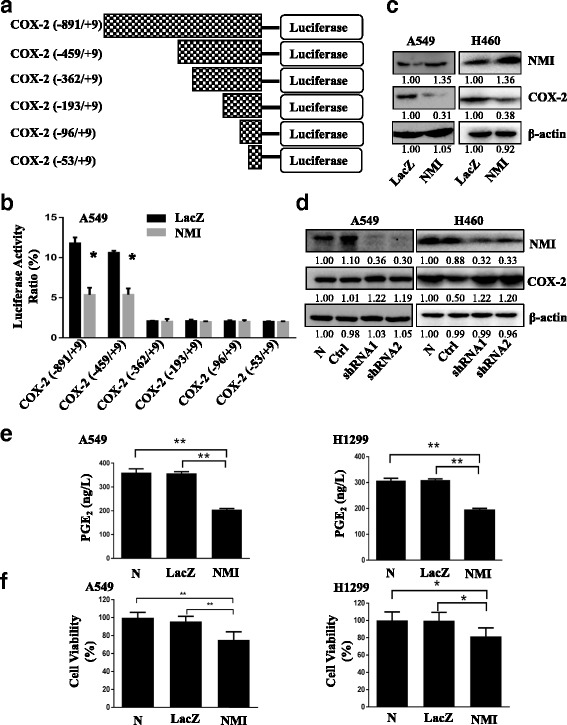
NMI regulated transcriptional activation of COX-2 and PGE2 production. **a** Schematic diagram of 6 different fragments of COX-2 promoter. **b** The activity of different fragments of COX-2 promoter was determined by luciferase reporter assay performed in A549 cells transfected with the control LacZ plasmid or the NMI overexpression plasmid. **c**, **d** Western blot and quantitative analysis were used to detected the expression of COX-2 in A549 and H460 cells with NMI overexpression (C) or NMI knockdown (D). **e** The PGE_2_ production level was measured in A549 and H1299 cell culture media at 72 h after transfection. **f** Cell viability was determined by MTT assay in A549 and H1299 cells

As NMI expression was inversely correlated with COX-2 expression in lung adenocarcinoma (Fig. 1a, b), we tested if NMI regulated COX-2 transcription through interacting with its promoter in lung adenocarcinoma cells. We inserted the full-length promoter of COX-2 (−891 to +9) as well as the truncated promoters COX-2 (−459 to +9), COX-2 (−362 to +9), COX-2 (−193 to +9), COX-2 (−96 to +9) and COX-2 (−53 to +9) into the luciferase reporter vector (Fig. 4a). Luciferase activity assay showed that overexpression of NMI in A549 cells significantly repressed promoters COX-2 (−891 to +9) and COX-2 (−459 to +9) (Fig. 4b). We further confirmed that overexpression of NMI in A549 and H460 cells caused decreased expression of COX-2 (Fig. 4c), while knockdown of NMI increased COX-2 expression in both A549 and H460 cells (Fig. 4d). In addition, overexpression of NMI in A549 and H1299 cells significantly reduced PGE2 production (Fig. 4e), which was consistent with inhibited COX-2 expression. Cell viability assay showed that overexpression of NMI caused significant cell death in A549 and H1299 cells (Fig. 4f), which might be a result of apoptosis induced by COX-2 inhibition [46–48].

### NMI regulated COX-2 transcription through p300

**Fig. 5:**
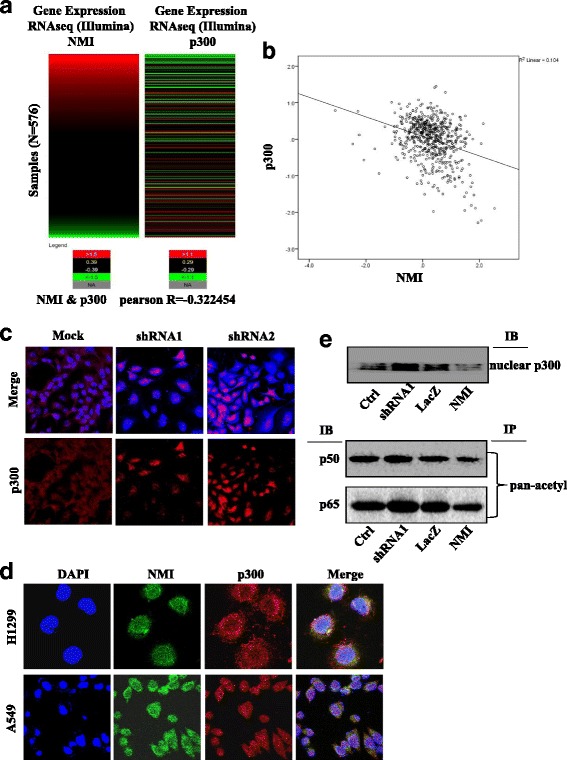
NMI regulated COX-2 transcription through p300. **a** TCGA data was analyzed for the correlation of NMI and p300 gene expression levels (*n* = 576). *Red* = high, *black* = medium, *green* = low. **b** Negative correlation in the expression of NMI and p300 was determined in TCGA database. **c** The expression of p300 in A549 cells with NMI knockdown was detected by immunofluorescence. **d** The subcellular localization of NMI and p300 in H1299 and A549 cells were examined by confocal microscopy. More than 100 cells were inspected per experiment, and cells with typical morphology were presented. **e** The expression of the nuclear p300 (*upper panel*), and the level of the nuclear p50 and p65 pulled down by the anti-pan-acetyl antibody (i.e. acetylated p50 and p65 in the nucleus, *lower panel*) were detected by western blot 48 h after transfection

**Fig. 6:**
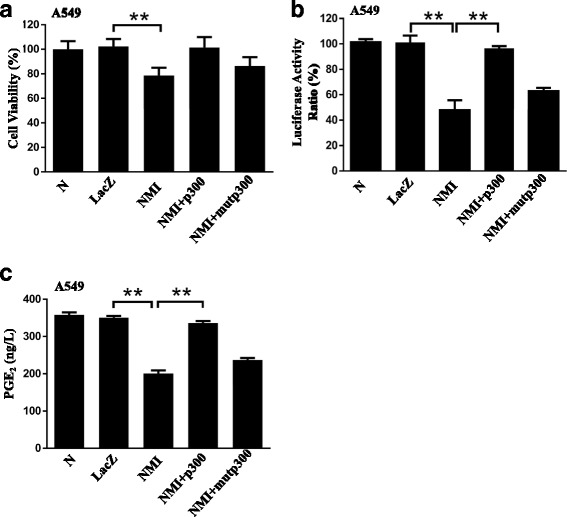
Overexpression of p300 reversed the cellular effects caused by NMI overexpression. **a** Cell viability was measured by MTT assay in A549 cells transfected with LacZ control plasmid, NMI overexpression plasmid, NMI + p300 overexpression plasmids, or NMI + HAT domain-deleted p300 overexpression plasmids. **b** COX-2 promoter activity was determined by luciferase reporter assay in A549 cells transfected with NMI and p300 overexpression plasmids. **c** PGE_2_ level was detected in the culture media of A549 cells transfected with NMI and p300 overexpression

p300 is an important transcripional co-activator for COX-2 transcription and has been shown to acetylate COX-2 promoter-bound transactivators such as NF-κB to activate COX-2 expression [50]. To determine whether p300 plays a role in the NMI-mediated regulation of COX-2 transcription, we first examined the interaction between p300 and NMI. We queried the NCI-Cancer Genome Atlas (TCGA) data base for lung adenocarcinoma samples (*n* = 576), and found that the expression of NMI and p300 were inversely correlated, with Pearson correlation coefficient of −0.322 and *P* < 0.05 (Fig. 5a, b). Immunofluorescence assay showed that the expression of p300 increased when NMI was knocked down (Fig. 5c). Next, we analyzed the subcellular localization of p300 and NMI in A549 and H1299 cells by immunofluorescence staining, and found that NMI and p300 co-localized in the cells (Fig. 5d). Furthermore, we observed in A549 cells that nuclear p300 was decreased when NMI was overexpressed, but was increased when NMI was knocked down (Fig. 5e, upper panel). Consistently, the acetylation level of p50 and p65 NF-κB, the nuclear targets of p300, was decreased in the NMI-overexpressed cells, but increased in the NMI-knockdown cells (Fig. 5e, lower panel). These results indicated that NMI might repress COX-2 expression by inhibiting p300. To confirm these results, we overexpressed p300 together with NMI in lung cancer cell line A549. The results showed that overexpression of p300, but not its histone acetyl transferase (HAT) domain deletion mutant (DHAT), was able to rescue the decreases in cell viability caused by NMI overexpression (Fig. 6a). Consistently, p300 transfection in the NMI-overexpressed A549 cells also rescued the transcriptional inhibition of COX-2 promoter (Fig. 6b) and PGE2 production (Fig. 6c).

### Overexpression of NMI suppressed tumor growth in a xenograft mouse model

**Fig. 7:**
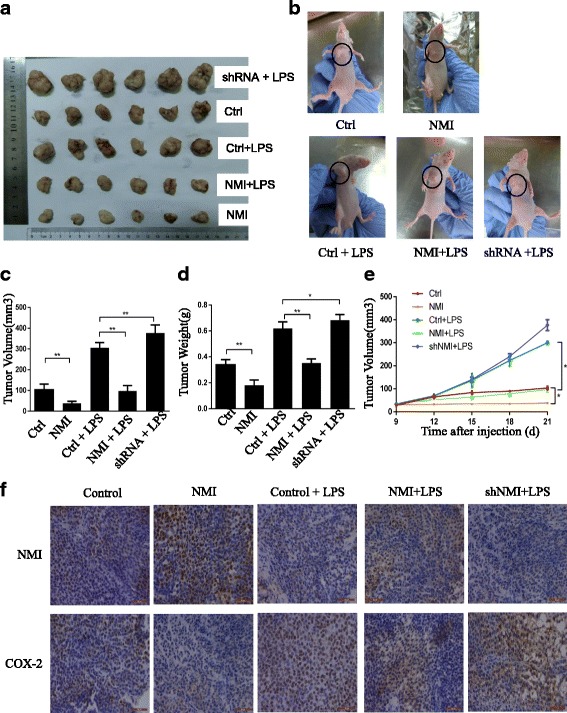
Overexpression of NMI suppressed tumor growth in a xenograft mouse model. Human A549 cells transfected respectively with (1) control plasmid (Ctrl), (2) NMI overexpression plasmid (NMI), (3) Ctrl + LPS, (4) NMI + LPS, (5) NMI shRNA + LPS (shRNA + LPS) were injected into the right flank of nude mice (*n* = 6 per group). **a** Tumors from the mice after sacrifice. **b** Representative images of the xenograft-bearing mice. **c** Tumor volume of each mouse at the time of sacrifice was measured. **d** Tumor weight of each mouse at the end of the experiment was measured. **e** Tumor volume of each mouse was recorded every three days during the course of the experiment. **f** The expression of NMI and COX-2 in xenografts were detected by HE and immunohistochemical staining (400 × magnification)

We validated the the suppression of NMI on lung cancer cell growth via the COX-2 in a xenograft mouse model. The A549 cells stably expressing NMI were injected into nude mice (flank) to establish human tumor xenograft. The results showed that overexpression of NMI significantly reduced tumor volume (Fig. 7a–e) and tumor weight (Fig. 7d). Lipopolysaccharide (LPS) is a COX-2 inducer and has been shown to be able to promote tumor growth. We found that LPS treatment could overcome the tumor growth inhibition caused by NMI overexpression (Fig. 7a-e). More importantly, knockdown of NMI combined with LPS treatment increased both the volume and weight of tumors, compared with the control group treated with LPS treatment alone (Fig. 7a-e). Moreover, immunohistochemical staining showed that NMI expression was negatively correlated with COX-2 expression in xenograft tumor tissues (Fig. 7f). These data further supported that NMI inhibited tumor cell growth through the suppression of COX-2 signaling in lung adenocarcinoma cells.

### High NMI expression was correlated with low COX-2 expression and predicted favorable prognosis in NSCLC patients

**Fig. 8:**
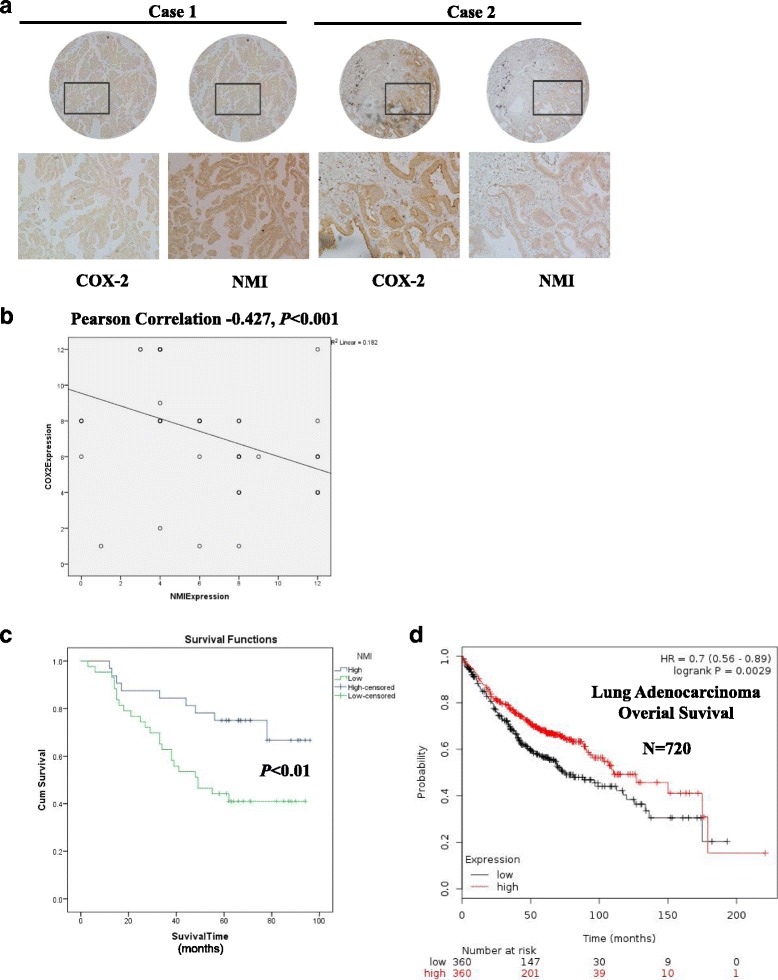
High NMI expression was correlated with low COX-2 expression and predicted favorable prognosis in NSCLC patients. **a** Representative images of the immunohistochemical staining of NMI and COX-2 in human lung adenocarcinoma tissues. **b** NMI expression was negatively correlated with COX-2 expression in lung adenocarcinoma samples (Pearson’s correlation test, *R* = −0.427, *P* < 0.001). **c** Kaplan-Meier analysis of the overall survival of 75 lung adenocarcinoma patients with different NMI expression. **d** Kaplan-Meier analysis of the overall survival of 720 lung adenocarcinoma patients with different NMI expression in KM PLOTTER database

We next evaluated the expression of NMI and COX-2 in tumor samples from 75 NSCLC patients by immunohistochemical (IHC) staining of tissue microarray. The expression of NMI and COX-2 in each IHC sample was classified either as high (score > 6) or low (score ≤ 6), using the median of the IHC scores as the cut-off value. We found that the expression of COX-2 was negatively correlated with NMI expression (Fig. 8a, b) (Pearson correlation coefficient − 0.427, *P* < 0.001).

Kaplan-Meier analyses of the overall survival revealed that the high NMI expression group (IHC staining with strong and moderate expression) had a better prognosis than the low NMI group (IHC staining with weak and negative expression) (*P* < 0.01) (Fig. 8c). In addition, we further assessed the correlation between NMI expression and lung adenocarcinoma patients’ overall survival in a 720 lung adenocarcinoma patient cohort from the KMPLOT database (http://www.kmplot.com) by Kaplan-Meier analyses, and found that NMI high expression patients had significantly longer overall survival (OS) than NMI low expression patients in the 720 lung adenocarcinoma patient (Fig. 8d).

For the 75 lung adenocarcinoma patients in our study, the correlation between NMI expression and clinicopathologic features was further evaluated and summarized in Table 1. The data showed no significant correlation between NMI downregulation and patient’s gender (*P* = 0.351, χ2 tests), age (≥60 vs. <60 years old) (*P* = 0.816, χ2 tests), T classification (*P* = 0.443, χ2 tests) and N classification (*P* = 0.451, χ2 tests). We next used a univariate analysis to evaluate associations between patient prognosis and several clinicopathologic factors including NMI expression (high vs. low), gender (male vs. female), age (≥60 vs. <60 years old), T stage (T3-T4 vs.T1) and N stage (N2 + N3 + Nx vs. N0 + N1). The results showed that high NMI expression was significantly associated with inferior overall survival (*P* = 0.008, HR = 0.378) (Table 2). Multivariate analysis also indicated that high NMI level was an independent prognostic factor for overall survival in patients with lung adenocarcinomas (Table 2). Taken together, all these findings suggested that high expression of NMI indicated better prognosis of lung adenocarcinoma patients.

**Table 1:** Association of NMI expression with patients’ clinicopathological features in human lung adenocarcinoma (*n* = 75)

	Total adenocarcinoma (*N* = 75)	NMI low expression (*N* = 43)	NMI high expression (*N* = 43)	*p*
Gender				
Male	39	20 (51.3%)	19 (48.7%)	0.351
Female	36	23 (63.9%)	13 (36.1%)	
Age at diagnosis				
< 60	43	24 (55.8%)	19 (44.2%)	0.816
≥ 60	32	19 (59.4%)	13 (40.6)	
pT factor				
T1	21	9 (42.9%)	12 (57.1%)	0.443
T2	42	25 (59.5%)	17 (40.5%)	
T3 + T4	12	6 (50%)	6 (50%)	
pN factor				
N0 + N1	52	28 (53.8%)	24 (46.2%)	0.4513
N2 + N3 + NX	23	15 (65.2%)	8 (34.8%)

**Table 2:** Cox proportional hazards model analysis of various prognostic factors in patients with lung adenocarcinoma

	Hazard ratio	95% Confidence interval	Unfavorable/Favorable	*p*
Univariate analysis				
NMI	0.378	(0.176, 0.813)	Low/High	0.008
Gender	1.069	(0.545, 2.097)	Male/Female	0.847
Age, y	0.795392	(0.398, 1.589)	<60y/≥60y	0.517
pT factor	1.382	(0.835, 2.289)	T3 + T4/T1	0.209
pN factor	1.39	(0.687, 2.813)	N2 + N3 + NX/N0 + N1	0.359
Multivariate analysis				
NMI	0.370	(0.171, 0.800)	Low/High	0.011
pT factor	1.441	(0.840, 2.474)	T3 + T4/T1	0.185
pN factor	1.391	(0.577, 2.457)	N2 + N3 + NX/N0 + N1	0.637

## Discussion

The data presented here have demonstrated that NMI functions as a COX-2 regulator in human NSCLC cells. Up-regulated expression of COX-2 in various cancers [37] and loss of NMI expression in advanced breast cancer [15] have been documented independently. Our data suggest that NMI plays a crucial role in the regulation of COX-2 transactivation. Overexpression of NMI resulted in the decrease of COX-2 expression and PGE2 production. Conversely, knockdown of NMI promoted COX-2 expression and PGE2 production. Overexpression of NMI in lung adenocarcinoma cells also inhibited cell proliferation and migration. In addition, we found that NMI expression was lower in lung cancer tumor tissues compared to the adjacent normal tissues. More importantly, human tissue arrays revealed that overexpression of NMI indicated a better prognosis in lung adenocarcinoma patients. Consistent with our results, recent reports have also shown that NMI inhibited tumor development [18]. Devine et al. found that NMI protein expression is higher in early stage breast cancer than in later stages, with the lowest expression observed in patients with stage IV breast cancer. Moreover, NMI mRNA expression is inversely related to the grade of clinical breast cancer specimens, indicating that the loss of NMI correlates with progression to aggressive disease [15]. Thus, loss of NMI may be associated with the progression of cancer.

In our study, we found that there was a significant negative correlation (Pearson correlation coefficient − 0.322, *P* = 0.011) between the expression of NMI and p300 in lung adenocarcinoma in TCGA database which contains 576 samples. In our own cohort containing 75 paired NSCLC normal and tumor samples, we also revealed a negative correlation between the expression of NMI and COX-2. More importantly, down-regulation of NMI was associated with poor survival of patients, which is further validated using the public database containing 720 patients. These data suggest NMI may function as a tumor suppressor in NSCLC. However, in this study, we used only a small volume of samples (75 totally). For the T stage, most of the patients (42 patients) were T2. Thus, we compared the survival between T3/T4 versus T1 groups. We could see a trend towards poorer survival for the late T stage group (the left figure). For the N stage, N0 and N1 samples were merged (52 totally) and compared to the rest of samples (23 totally). We could see a similar trend towards poorer survival in the late N stage group (the right figure). Unfortunately, the difference in both comparisons were in-significant. This may be caused by the small sample volumes, in the future study, we will increase sample sizes to solve the problem.

We also found that the survival curves of NMI high and low expression groups crossed at 170 months in a 720 lung adenocarcinoma patient cohort from the KMPLOT database This mabe be due to several outlier patients who live for quite long time, which is not usually seen for lung cancer. A plausible explanation may be that those patients are actually died of other causes. As a result, the two curves tend to overlap after long time followup. If looking at the earlier parts of the curves, such as five years or ten years, the two groups are actually widely separated.

In our current study, we didn’t observe a perfect inverse correlation between NMI and COX-2 in our randomly selected 4 cell lines. Two cell lines H322 and H1299 showed low NMI expression but negative COX-2 expression. This may reflect the complex regulatory network of COX-2 in cancer cells. Actually, many other factors, such as IL-1 and HIF1α, have been identified to regulate COX-2 expression. It’s possible that these unknown factors may interfere with NMI/COX-2 signaling. However, in a much larger cohort of clinical samples, we observed a statistically significant inverse correlation between NMI and COX-2.

As a common transcriptional coactivator, p300 has been proved to be involved in the regulation of COX-2 gene expression. We therefore hypothesized that p300 interact with NMI, and they have opposite effect on the regulation of COX-2 expression. Our data supported the hypothesis that overexpression of NMI reduced nuclear p300 localization and the acetylation of its targets p50 and p65 NF-kB. Moreover, the elevated p300 level could rescue the inhibition of COX-2 expression and cell proliferation in NMI overexpressed cells. These data indicate p300 may play an important role in the NMI-mediated regulation of COX-2 expression in lung adenocarcinoma cells. Further studies are needed to confirm the detailed molecular mechanisms by which NMI regulates COX-2 expression.

## Conclusions

In summary, we have discovered that NMI plays an important role in the regulation of COX-2 expression and tumor growth in lung adenocarcinoma, and high expression of NMI suggests to a better prognosis in human adenocarcinoma. Our study therefore suggests that the NMI/COX-2 signaling pathway is a potential prognostic biomarker for lung cancers.

## Abbreviations

AC: Qadenocarcinoma
EGFR: Epidermal growth factor receptor
EML4-ALK: Echinoderm microtubule-associated protein-like 4-anaplastic lymphoma kinase
EMT: Epithelial-mesenchymal-transition
HAT: Histone acetyltransferase
NMI: (N-myc (and STAT) interactor
NSCLC: Non-small-cell lung cancer
PGE2: Prostaglandin E2
